# Supplementary material for: Plasma concentrations of nirmatrelvir and molnupiravir required for inhibition of SARS-CoV-2 replication differ between rhesus macaques and humans
Source: Antimicrob Agents Chemother. 2026 Aug 5;70(9):e00307-26. doi: 10.1128/aac.00307-26 (PMC13712808; doi:10.1128/aac.00307-26)
Supplement: Supplementary file 1 [file aac.00307-26-s0001.docx]

**Supplementary material**

**Plasma nirmatrelvir and molnupiravir concentrations required for inhibition of SARS-CoV-2 replication differ between rhesus macaques and humans**

Ugo Avila-Ponce de Leon^1^, Shadisadat Esmaeili, Katherine Owens^1^, Joshua T. Schiffer^1,2^

^1^ Vaccine and Infectious Diseases Division, Fred Hutchinson Cancer Research Center, 1100 Fairview Ave., Seattle, WA, 98122, USA.

^2^ Department of Medicine, University of Washington, 1959 NE Pacific St., Seattle, WA, 98195, USA.

**Table S1. All tested models against lung and nasal viral load data. AIC and LL values written in red are for nasal, and in blue are the values for lung.** The selected model is bolded in the bottom row.

| **Model** | **Equations** | **Free parameters** | **Fixed parameters** | **LL / AIC** |
| --- | --- | --- | --- | --- |
| Target cell limited | $\frac{dS}{dt}= - \beta SVi$  $\frac{dI}{dt}= \beta SVi- \delta I$  $\frac{dVi}{dt}=\pi(1-\mu)I-cVi$  $\frac{dVni}{dt}=\pi\mu I-cVni$ | $\delta$  $\pi$  $\mu$  $c$ |  | 6371.63/6387.63  5647.83/5663.83 |
| Refractory cells | $\frac{dS}{dt}= -\beta SVi- \phi IS+ \rho R$  $\frac{dR}{dt}= \phi IS- \rho R$  $\frac{dI}{dt}= \beta SVi- \delta I$  $\frac{dVi}{dt}=\pi(1-\mu)I-cVi$  $\frac{dVni}{dt}=\pi\mu I-cVni$ | $\delta$  $\pi$  $\mu$  $c$  $\phi$  $\rho$ | $\beta$=10^-3^ | 4289.36/4313.36  3422.94/3446.94 |
| Eclipse target cell limited | $\frac{dS}{dt}= - \beta SVi$  $\frac{dIe}{dt}= \beta SVi- kIe$  $\frac{dIp}{dt}= kIe- \delta Ip$  $\frac{dVi}{dt}=\pi(1-\mu)Ip-cVi$  $\frac{dVni}{dt}=\pi\mu Ip-cVni$ | $\delta$  $\pi$  $\mu$  $c$  $k$ | $\beta$=10^-3^ | 6363.81/6379.81  5698.02/5714.02 |
| **Eclipse refractory** | $\frac{\boldsymbol{dS}}{\boldsymbol{dt}}\boldsymbol{= -\beta SVi- \phi IpS+ \rho R}$  $\frac{\boldsymbol{dR}}{\boldsymbol{dt}}\boldsymbol{= \phi IpS- \rho R}$  $\frac{\boldsymbol{dIe}}{\boldsymbol{dt}}\boldsymbol{= \beta SVi- kIe}$  $\frac{\boldsymbol{dIp}}{\boldsymbol{dt}}\boldsymbol{= kIe- \delta Ip}$  $\frac{\boldsymbol{dVi}}{\boldsymbol{dt}}\boldsymbol{=\pi(1-}\boldsymbol{\mu}\boldsymbol{)Ip-cVi}$  $\frac{\boldsymbol{dVni}}{\boldsymbol{dt}}\boldsymbol{=\pi}\boldsymbol{\mu}\boldsymbol{Ip-cVni}$ | $\boldsymbol{\delta}$  $\boldsymbol{\pi}$  $\boldsymbol{\mu}$  $\boldsymbol{c}$  $\boldsymbol{\phi}$  $\boldsymbol{\rho}$  $\boldsymbol{k}$ | $\boldsymbol{\beta}$**=10^-3^** | **4425.27/4449.27**  **3419.49/3443.49** |
| Target cell limited / TCID50 cleared with infected cells | $\frac{dS}{dt}= - \beta SVi$  $\frac{dI}{dt}= \beta SVi- \delta I$  $\frac{dVi}{dt}=\pi(1-\mu)I-\delta Vi$  $\frac{dVni}{dt}=\pi\mu I-cVni$ | $\delta$  $\pi$  $\mu$  $c$ | $\beta$=10^-3^ | 6531.43/6547.43  5782.02/5798.02 |
| Refractory cells / TCID50 cleared with infected cells | $\frac{dS}{dt}= -\beta SVi- \phi IS+ \rho R$  $\frac{dR}{dt}= \phi IS- \rho R$  $\frac{dI}{dt}= \beta SVi- \delta I$  $\frac{dVi}{dt}=\pi(1-\mu)I-\delta Vi$  $\frac{dVni}{dt}=\pi\mu I-cVni$ | $\delta$  $\pi$  $\mu$  $c$  $\phi$  $\rho$ | $\beta$=10^-3^ | 4818.58/4842.58  3419.01/3443.01 |
| Eclipse target cell limited / TCID50 cleared with infected cells | $\frac{dS}{dt}= - \beta SVi$  $\frac{dIe}{dt}= \beta SVi- kIe$  $\frac{dIp}{dt}= kIe- \delta Ip$  $\frac{dVi}{dt}=\pi(1-\mu)Ip-\delta Vi$  $\frac{dVni}{dt}=\pi\mu Ip-cVni$ | $\delta$  $\pi$  $\mu$  $c$  $k$ | $\beta$=10^-3^ | 6548.86/6564.86  5829.13/5845.13 |
| Eclipse refractory / TCID50 cleared with infected cells | $\frac{dS}{dt}= -\beta SVi- \phi IpS+ \rho R$  $\frac{dR}{dt}= \phi IpS- \rho R$  $\frac{dIe}{dt}= \beta SVi- kIe$  $\frac{dIp}{dt}= kIe- \delta Ip$  $\frac{dVi}{dt}=\pi(1-\mu)Ip-\delta Vi$  $\frac{dVni}{dt}=\pi\mu Ip-cVni$ | $\delta$  $\pi$  $\mu$  $c$  $\phi$  $\rho$  $k$ | $\beta$=10^-3^ | 4435.86/4463.86  3391.86/3419.86 |

**Table S2. Estimated parameter values for fit to untreated animals.** Parameter values from fits to the 5 animals from the Rosenke study (black) are compared to fits from 43 control animals (red). Separate tables are shown for nasal and BAL fits.

**Nasal**

| **ID** | $\boldsymbol{\beta}$ | $\boldsymbol{\delta}$ | $\boldsymbol{\mu}$ | $\boldsymbol{p}$ | $\boldsymbol{\rho}$ | $\boldsymbol{\varphi}$ |
| --- | --- | --- | --- | --- | --- | --- |
| **VF1** | $\boldsymbol{10}^{\boldsymbol{-3.02}}\left( \boldsymbol{10}^{\boldsymbol{-3.33}} \right)$ | **2 (1.99)** | **0.0094(0.011)** | $\boldsymbol{10}^{\boldsymbol{1.67}}\left( \boldsymbol{10}^{\boldsymbol{1.87}} \right)$ | $\boldsymbol{10}^{\boldsymbol{0.53}}\left( \boldsymbol{10}^{\boldsymbol{-1.89}} \right)$ | $\boldsymbol{10}^{\boldsymbol{-5.34}}\left( \boldsymbol{10}^{\boldsymbol{-5.02}} \right)$ |
| **VF2** | $\boldsymbol{10}^{\boldsymbol{-2.92}}\left( \boldsymbol{10}^{\boldsymbol{-3.06}} \right)$ | **2.89(2.78)** | **0.0013(0.0013)** | $\boldsymbol{10}^{\boldsymbol{2.36}}\left( \boldsymbol{10}^{\boldsymbol{2.48}} \right)$ | $\boldsymbol{10}^{\boldsymbol{-0.21}}\left( \boldsymbol{10}^{\boldsymbol{-2.04}} \right)$ | $\boldsymbol{10}^{\boldsymbol{-6.41}}\left( \boldsymbol{10}^{\boldsymbol{-5.18}} \right)$ |
| **VF3** | $\boldsymbol{10}^{\boldsymbol{-2.87}}\left( \boldsymbol{10}^{\boldsymbol{-3.02}} \right)$ | **3.14(2.92)** | **0.0042(0.0038)** | $\boldsymbol{10}^{\boldsymbol{1.86}}\left( \boldsymbol{10}^{\boldsymbol{2.02}} \right)$ | $\boldsymbol{10}^{\boldsymbol{-0.44}}\left( \boldsymbol{10}^{\boldsymbol{-2.03}} \right)$ | $\boldsymbol{10}^{\boldsymbol{-6.13}}\left( \boldsymbol{10}^{\boldsymbol{-4.94}} \right)$ |
| **VF4** | $\boldsymbol{10}^{\boldsymbol{-2.93}}\left( \boldsymbol{10}^{\boldsymbol{-2.77}} \right)$ | **2.64(3.79)** | **0.015(0.015)** | $\boldsymbol{10}^{\boldsymbol{1.43}}\left( \boldsymbol{10}^{\boldsymbol{1.25}} \right)$ | $\boldsymbol{10}^{\boldsymbol{-0.011}}\left( \boldsymbol{10}^{\boldsymbol{-0.16}} \right)$ | $\boldsymbol{10}^{\boldsymbol{-2.24}}\left( \boldsymbol{10}^{\boldsymbol{-2.74}} \right)$ |
| **VF5** | $\boldsymbol{10}^{\boldsymbol{-2.92}}\left( \boldsymbol{10}^{\boldsymbol{-2.87}} \right)$ | **3.15(4.1)** | **0.0018(0.0019)** | $\boldsymbol{10}^{\boldsymbol{2.99}}\left( \boldsymbol{10}^{\boldsymbol{3.09}} \right)$ | $\boldsymbol{10}^{\boldsymbol{-0.53}}\left( \boldsymbol{10}^{\boldsymbol{-0.57}} \right)$ | $\boldsymbol{10}^{\boldsymbol{-3.25}}\left( \boldsymbol{10}^{\boldsymbol{-3.16}} \right)$ |

**BAL**

| **ID** | $\boldsymbol{\beta}$ | $\boldsymbol{\delta}$ | $\boldsymbol{\mu}$ | $\boldsymbol{p}$ | $\boldsymbol{\rho}$ | $\boldsymbol{\varphi}$ |
| --- | --- | --- | --- | --- | --- | --- |
| **VF1** | $\boldsymbol{10}^{\boldsymbol{-2.34}}\left( \boldsymbol{10}^{\boldsymbol{-2.33}} \right)$ | **5.12(4.89)** | **0.0012(0.0014)** | $\boldsymbol{10}^{\boldsymbol{2.15}}\left( \boldsymbol{10}^{\boldsymbol{2.11}} \right)$ | $\boldsymbol{10}^{\boldsymbol{-2.52}}\left( \boldsymbol{10}^{\boldsymbol{-2.99}} \right)$ | $\boldsymbol{10}^{\boldsymbol{-5.05}}\left( \boldsymbol{10}^{\boldsymbol{-5.07}} \right)$ |
| **VF2** | $\boldsymbol{10}^{\boldsymbol{-2.32}}\left( \boldsymbol{10}^{\boldsymbol{-2.8}} \right)$ | **0.85(0.92)** | **0.15(0.15)** | $\boldsymbol{10}^{\boldsymbol{0.69}}\left( \boldsymbol{10}^{\boldsymbol{0.96}} \right)$ | $\boldsymbol{10}^{\boldsymbol{0.46}}\left( \boldsymbol{10}^{\boldsymbol{-1.72}} \right)$ | $\boldsymbol{10}^{\boldsymbol{-4.83}}\left( \boldsymbol{10}^{\boldsymbol{-4.28}} \right)$ |
| **VF3** | $\boldsymbol{10}^{\boldsymbol{-2.33}}\left( \boldsymbol{10}^{\boldsymbol{-2.18}} \right)$ | **3.76(2.61)** | **0.0054(0.0055)** | $\boldsymbol{10}^{\boldsymbol{1.49}}\left( \boldsymbol{10}^{\boldsymbol{1.35}} \right)$ | $\boldsymbol{10}^{\boldsymbol{-1.12}}\left( \boldsymbol{10}^{\boldsymbol{-1.31}} \right)$ | $\boldsymbol{10}^{\boldsymbol{4.51}}\left( \boldsymbol{10}^{\boldsymbol{-4.35}} \right)$ |
| **VF4** | $\boldsymbol{10}^{\boldsymbol{-2.33}}\left( \boldsymbol{10}^{\boldsymbol{-2.59}} \right)$ | **0.67(0.64)** | **0.01(0.01)** | $\boldsymbol{10}^{\boldsymbol{1.42}}\left( \boldsymbol{10}^{\boldsymbol{1.58}} \right)$ | $\boldsymbol{10}^{\boldsymbol{0.57}}\left( \boldsymbol{10}^{\boldsymbol{-1.79}} \right)$ | $\boldsymbol{10}^{\boldsymbol{-4.78}}\left( \boldsymbol{10}^{\boldsymbol{-4.71}} \right)$ |
| **VF5** | $\boldsymbol{10}^{\boldsymbol{-2.31}}\left( \boldsymbol{10}^{\boldsymbol{-1.9}} \right)$ | **3.58(2.61)** | **0.0012(0.0012)** | $\boldsymbol{10}^{\boldsymbol{2.37}}\left( \boldsymbol{10}^{\boldsymbol{2.27}} \right)$ | $\boldsymbol{10}^{\boldsymbol{-1.1}}\left( \boldsymbol{10}^{\boldsymbol{-1.34}} \right)$ | $\boldsymbol{10}^{\boldsymbol{-4.79}}\left( \boldsymbol{10}^{\boldsymbol{-4.6}} \right)$ |

**Table S3. Comparison of parameters between viral variants and anatomic compartments.** Blue is the value of the washington strain WA.1; red is the delta variant and green is Omicron.

| Nasal | | | |
| --- | --- | --- | --- |
| Parameters | Q1 | Median | Q3 |
| $\beta\log_{10} \left( {PFU}/{ml} \right)^{-1}{day}^{-1}$ | -3.11  -3.05  -3.16 | -2.85  -3.02  -2.94 | -2.71  -2.87  -2.36 |
| $\delta\left( {days}^{-1} \right)$ | 1.39  2.78  1.88 | 2.52  2.92  3.03 | 4.37  3.79  3.96 |
| $\mu$ | 0.0055  0.019  0.005 | 0.0075  0.0038  0.008 | 0.0089  0.0106  0.015 |
| $p \left( \log_{10} {days}^{-1} \right)$ | 1.28  1.87  1.51 | 1.58  2.01  1.74 | 2.05  2.48  1.83 |
| $\varphi\left( \log_{10} {days}^{-1} \right)$ | -5.03  -5.01  -5.32 | -4.50  -4.93  -4.82 | -4.23  -3.16  -4.27 |
| $\rho\left( \log_{10} {days}^{-1} \right)$ | -2.67  -2.02  -3.01 | -1.96  -1.89  -1.19 | -1.11  -0.57  -0.78 |
| BAL | | | |
| Parameters | Q1 | Median | Q3 |
| $\beta$ $\log_{10} \left( {PFU}/{ml} \right)^{-1}{day}^{-1}$ | -2.58  -2.58  -2.12 | -2.37  -2.33  -1.89 | -1.97  -2.18  -1.5 |
| $\delta$ $\left( {days}^{-1} \right)$ | 1.65  0.91  1.82 | 3.02  2.61  2.20 | 4.96  2.61  3.01 |
| $\mu$ | 0.011  0.0013  0.017 | 0.013  0.054  0.0288 | 0.02  0.0103  0.037 |
| $p \left( \log_{10} {days}^{-1} \right)$ | 0.82  1.35  0.749 | 1.04  1.57  1.37 | 1.20  2.10  2.007 |
| $\varphi\left( \log_{10} {days}^{-1} \right)$ | -5.28  -4.71  -4.75 | -4.62  -4.59  -4.35 | -4.05  -4.35  -4.22 |
| $\rho\left( \log_{10} {days}^{-1} \right)$ | -2.77  -1.78  -3.06 | -1.64  -1.71  -1.43 | -0.46  -1.33  -0.0405 |

**Table S4. Parameters for PK models for molnupiravir and nirmatrelvir in non-human primates.**

| Parameters | Molnupiravir | Nirmatrelvir |
| --- | --- | --- |
| $k_{a}$ $\left( {day}^{-1} \right)$ | 16.48 | 57.89 |
| $k_{LP}$ $\left( {day}^{-1} \right)$ | 0.71 | 1.21 |
| $k_{PL}$ $\left( {day}^{-1} \right)$ | 2.36 | 2.81 |
| $k_{cl}$ $\left( {day}^{-1} \right)$ | 13.55 | 30.81 |
| $Vol$ $\left( ml \right)$ | 92569.9 | 25058.3 |

**Figure S1. Observed viral loads during SARS-CoV-2 infection without treatment (n=43), 38 WA1 and 5 delta.** **(A-B)** Nasal viral loads at days 1, 2 and 4. **(C-D)** Lung viral loads at days 1, 2 and 4. **(A, C)** SARS-CoV-2 RNA levels and **(B, D)** SARS-CoV-2 TCID50 levels. Wilcoxon test was carried out for the comparison between variants in **A and C** *p<0.05, **p<0.01 , ***, p<0.001.

**Figure S2. Model fits to SARS-CoV-2 nasal compartment viral loads in untreated RM.** Fits for all 43 individuals were evaluated to estimate parameters. Triangles and line in color purple are TCID50 values fitted for the data, meanwhile circles and line in color pink are for PCR data. The dashed black line in all panels is the limit of detection. Hollow triangles are undetectable values.

**Figure S3. Model fits to SARS-CoV-2 BAL compartment viral loads in untreated R.*,*** Fits for all 43 individuals were evaluated to estimate parameters. Triangles and line in color purple are TCID50 values fitted for the data, meanwhile circles and line in color pink are for PCR data. The dashed black line in all panels is the limit of detection*. Hollow triangles* are undetectable *values.*

**Figure S4. Pharmacokinetic (PK) model of plasma concentration of nirmatrelvir.** PK model fits individual nirmatrelvir concentration in plasma for 18 rhesus macaques all given two doses daily for 16 days. The respective doses were (A) 20 mg/kg (B) 50 mg/kg, or (C) 300 mg/kg.

**Figure S5**. **Pharmacokinetic (PK) model of plasma concentration of nirmatrelvir between day 0 and 1.** PK model fits individual of nirmatrelvir concentration in plasma for 18 rhesus macaques all given two doses daily for 16 days. The respective doses were (A) 20 mg/kg (B) 50 mg/kg or (C) 300 mg/kg.

**Figure S6. Pharmacokinetic (PK) model of plasma concentration of nirmatrelvir between day 15 and 16.** PK model fits individual of nirmatrelvir concentration in plasma for 18 rhesus macaques all given two doses daily for 16 days. The respective doses were (A) 20 mg/kg (B) 50 mg/kg or (C) 300 mg/kg.

**Figure S7**. **Visual predictive check (VPC) for nirmatrelvir in both compartments.** (A) VPC and observed data points for nirmatrelvir impact in nasal compartment and (B) VPC in BAL compartments by PCR (A and C) and TCID50 (B and D). Black solid or dashed line are the observed percentiles, meanwhile the red and the blue lines are the simulated percentiles.

**Figure S8. Viral load area under the curve (AUC) and drop from baseline during nirmatrelvir treatment in rhesus macaques.** We calculated viral AUC (A and C) and decline during treatment (B and D) for TCID and viral PCR detected SARS-CoV-2 from nasal (A-B) and BAL (C-D) BAL samples in nirmatrelvir treated (red) versus untreated (blue) animals.

**Figure S9.** **Pharmacokinetic (PK) model of plasma concentration of Molnupiravir.** PK model fits individual of Molnupiravir concentration in plasma for 8 rhesus macaques all given a single dose of the drug of 100 mg/kg.

**Figure S10**. **Visual predictive check (VPC) for molnupiravir in both compartments.** (A) VPC and observed data points for molnupiravir impact in nasal compartment and (B) VPC in BAL compartments by PCR (A and C) and TCID50 (B and D). Black solid or dashed line are the observed percentiles, meanwhile the red and the blue lines are the simulated percentiles.

**Figure S11. Viral load area under the curve (AUC) and drop from baseline during molnupiravir treatment in rhesus macaques.** We calculated viral AUC (A and C) and decline during treatment (B and D) for TCID and viral PCR detected SARS-CoV-2 from nasal (A-B) and BAL (C-D) BAL samples in molnupiravir treated (red) versus untreated (blue) animals.
